# Word frequency and reading demands modulate brain activation in the inferior frontal gyrus

**DOI:** 10.1038/s41598-023-44420-z

**Published:** 2023-10-11

**Authors:** Abraham Sánchez, Manuel Carreiras, Pedro M. Paz-Alonso

**Affiliations:** 1 Basque Center On Cognition Brain and Language (BCBL), BCBL, Mikeletegi Pasealekua 69, 2, 20009 Donostia-San Sebastián, Spain; 2 University of the Basque Country (EHU/UPV), Donostia-San Sebastián, Spain; 3 IKERBASQUE, Basque Foundation for Science, Bilbao, Spain

**Keywords:** Cognitive neuroscience, Language, Reading, Neuroscience

## Abstract

Processing efficiency differs between high- and low-frequency words, with less frequent words resulting in longer response latencies in several linguistic behavioral tasks. Nevertheless, studies using functional MRI to investigate the word frequency effect have employed diverse methodologies and produced heterogeneous results. In this study, we examine the effect of word frequency through complementary analytical approaches and functional connectivity analyses. Furthermore, we examine whether reading demands, which have been shown to influence reading-related activation, modulate the effects of word frequency. We conducted MRI scanning on 54 healthy participants who performed two versions of a single-word reading task involving high- and low-frequency words: a low-level perceptual reading task and a high-level semantic reading task. The results indicate that word frequency influenced the activation of the *pars orbitalis* and *pars triangularis* of the inferior frontal gyrus, but only in the semantic reading task. Additionally, the ventral occipitotemporal cortex exhibited stronger regional activation during the semantic reading task compared to the perceptual reading task, with no effects of word frequency. Functional connectivity analyses demonstrated significant coupling among regions within both the dorsal and ventral reading networks, without any observable effects of word frequency or task. These findings were consistent across group- and individual-level analytical approaches. Overall, our results provide further support for the involvement of the inferior frontal gyrus in semantic processing during reading, as indicated by the effect of word frequency and the influence of reading demands, highlighting the role of the ventral reading network. These findings are discussed in line with their implications for lexical and pre-lexical reading processing.

## Introduction

What are the brain mechanisms that allow us to read more efficiently? How do our brains take advantage of repeated exposure to words? When we first read an unknown word, it requires an effortful processing of its basic orthographic and phonological units. Through repeated exposure to words as perceptual stimuli, we have the chance to encode and consolidate their phonological, morphological and syntactic features^1^. These new words are also linked to other words and concepts, and ultimately incorporated within a broader semantic network^2,3^. This process facilitates whole-word reading without the need for thorough perceptual processing, thus allowing for more efficient reading^4^.

In the reading domain, word frequency can be understood as a proxy for such repeated exposure. In fact, the word frequency effect (WFE) has been repeatedly reported in the neurobiology of reading literature: low-frequency words are typically harder to process than high-frequency words. Empirical evidence from behavioural studies has extensively shown faster response times for high-frequency as compared to low-frequency words, across many different tasks, such as word naming, lexical decision or semantic decision tasks^5^. At the neural level, a few functional Magnetic Resonance Imaging (fMRI) studies have specifically examined the WFE. In such studies, the WFE is defined as higher regional activation for low-frequency relative to high-frequency words, which has been taken as an indicator of more effortful processing. From this point on, the term WFE will refer to this functional effect. Most of these fMRI studies have located this effect in the inferior frontal gyrus (IFG). In some of these studies, the WFE extends to the anterior part of the IFG (i.e., *pars orbitalis* and *pars triangularis,* BA 47 and 45, respectively), while in others it is associated with the posterior IFG (i.e., *pars opercularis,* BA 44), or both. Other studies have also found the WFE in the ventral occipitotemporal cortex (vOTC). Table 1 summarises previous fMRI studies examining the WFE, their main findings, methodological conditions and sample sizes.

**Table 1:** Previous fMRI studies examining the WFE, main findings, reading tasks used and sample sizes.

Studies	Regions showing the WFE	Reading tasks	Sample sizes (N)
Chee et al.^6^	Left IFG (BA 44)	Silent reading vs. semantic judgements	16
Fiebach et al.^7^	Left IFG (BA 44, 45)	Lexical decision task	12
Chee et al.^8^	Left ACC (BA 32), IFG (BA 44, 45), ITC (BA 37)	Semantic judgement + 24 h Recognition	16
Joubert et al.^9^	Left IFG (BA 45, 47)	Silent reading	10
Kronbichler et al.^10^	Left IFG (BA 45, 47), vOTC (mid)	Silent reading	13
Carreiras et al.^11^	Left IFG (BA 44)	Lexical Decision vs Reading aloud	16
Graves et al.^12^	Left IFG (BA 45/47), vOTC (post), pSTG	Picture naming (overt)	59
Hauk et al.^13^	Bil. IFG (BA 45, 47), vOTC (ant)	Silent reading	21
Bruno et al.^14^	Left Precentral, IFG (BA 44, 45), vOTC, pSTG	Phonological lexical decision task	28
Carreiras et al.^15^	Bil. ACC/ IFG (BA 45, 47), Precuneus, SMA	Lexical decision task	20
Schuster et al.^16^	Left IFG (BA 45), vOTC	Silent sentence reading	56
Rundle et al.^17^	Left ITC (BA 37); vOTC	Silent reading + semantic catch trial	19

The heterogeneity of the tasks used has led to multiple interpretations about the nature of the WFE. While some authors interpret the WFE as phonological processing or retrieval during lexical search^7,15^, other authors have proposed that the WFE relies on deliberate access to semantics^6^. Here we propose that, if the WFE relies on phonological processes, then it would be mainly observable in regions along the *dorsal reading network* [i.e., IFG pars opercularis, superior temporal gyrus (STG), and/or inferior parietal cortex (IPC)] involved in mapping visual percepts onto the phonological structure of the language. In contrast, if the WFE relies on lexico-semantic processing, we predict that the WFE will mainly rely on the engagement of regions along the *ventral reading network* [i.e., IFG *pars triangularis,* IFG *pars orbitalis*, and/or the vOTC], involved in mapping orthographic-lexical stimuli to words as a whole^18,19^. Indeed, the studies defending the semantic interpretation have typically found the WFE in regions of the *ventral reading network*, whereas the studies defending the phonological interpretation have typically found the WFE to occur in regions along the *dorsal reading network* (see Table 1).

On the other hand, it has been indicated that the engagement of both the IFG and the vOTC could be modulated by top-down processes, such as those imposed by reading demands^17,20,21^. Some authors have proposed that, in the case of the IFG, lexical decisions^17^ or naming efforts^22^ might be amplifying the effects found in this region. In the case of the vOTC, the prolonged exposure time, combined with the demands of tasks like semantic judgement or lexical decision tasks, could increase engagement of this region during the processing of low-frequency words^16^. These contrasting interpretations are especially controversial in regard to the main theoretical accounts of the vOTC. Some authors have proposed that part of the vOTC is dedicated to the processing of visual word forms^23^. Nevertheless, the involvement of the vOTC has also been found in tasks that do not require the processing of visual word forms^24,25^. Whereas Deahaene & Cohen interpret this as a side effect of top-down processes, other authors suggest it illustrates the interaction between both top-down and bottom-up processes in the vOTC, and thus it is not exclusively dedicated to orthographic prelexical processing^20,26–28^. Recent studies have found that the vOTC could be further functionally and structurally segregated into a lexical and a perceptual division^29^, which supports the view that the vOTC participates in both lexical and prelexical processes^30^. As mentioned, some studies have analysed the influence of word frequency on the activation of the vOTC. The rationale behind this is that, if indeed the vOTC is involved in prelexical processing of visual word forms (i.e. computation of abstract letter strings), then activation in this region should not vary as a function of word frequency^10^. Nevertheless, a number of studies have found a WFE in the vOTC^10,12–14,16,17^, while others have failed to find such an effect^6–9,11,15^.

These mixed findings regarding the WFE and reading demands could be partially explained by the heterogeneity in the methodological characteristics of previous MRI studies. The studies on WFE have typically used diverse conditions and tasks, from silent reading to lexical decision. This fact has influenced the effects of frequency in the above-mentioned brain regions, which renders the results on the WFE hard to interpret. Moreover, the specific fMRI methodological procedures used in previous studies varied significantly, including the use of different protocols for multiple comparison corrections, or even the absence of any correction for multiple comparisons over the statistical significance thresholds. Furthermore, the majority of the previous fMRI studies on the WFE have focused on regional activation, whereas functional connectivity patterns that may underlie this effect have not been examined yet.

In light of these inconsistencies, here our main goal was to investigate the WFE in the activation profiles of regions along the ventral and dorsal reading networks using different complementary analytical approaches. We also aimed at examining the potential interaction between reading demands and word frequency in such functional patterns. For this, we employed two versions of a single-word reading task: a perceptual (low reading demand) task, and a semantic (high reading demand) task, enabling us to examine the influence of reading demands imposed by these two kinds of word reading tasks in the WFE. Lastly, we sought to examine whether there were any differentiable functional connectivity profiles among ventral and dorsal reading regions that are known to respond to word frequency and reading demands. In line with previous evidence and accounts regarding a division of labour among these two networks (e.g.,^31^), we expect to observe the WFE in regions of the lexico-semantic ventral reading network, such as the IFG and the vOTC, defined as higher regional activation in these areas for low-frequency words relative to high-frequency words. Moreover, we predict this effect will be stronger in the semantic task compared to the perceptual task. We also expect that such findings will replicate across different analytical approaches. In terms of functional connectivity, we expect the WFE to be associated with tighter functional connectivity within regions along the ventral reading network.

## Materials and methods

### Participants

The final sample of the study consisted of 54 right-handed native Spanish-speaking participants, with an average age of 29.3 years (SD = 6.88 years; 30 females). All participants had normal or corrected-to-normal vision, and no history of neurological or psychiatric illness. Of the initial 57 participants, one participant was excluded due to excessive head motion during scanning (see “fMRI data analysis” section below), and two participants were excluded due to the absence of responses to the catch (i.e., Go) trials in the fMRI tasks. Language proficiency was assessed using both objective and subjective measures. As an objective measure, we used an adapted Spanish version of the Boston Naming Test^32^. Participants also filled in a language proficiency self-rated questionnaire, in which they evaluated their own proficiency, as well as language exposure. Prior to taking part in the experiment, all participants gave written informed consent in compliance with the ethical regulations established by the BCBL Ethics Committee and the guidelines of the Helsinki Declaration. All participants received monetary compensation for their participation.

### Materials and procedure

The experimental design consisted of two single-word reading Go/No-Go tasks, one perceptual (low reading demand) and the other semantic (high reading demand). In both tasks, all participants were visually presented with character strings in their native language (i.e., Spanish) that could be words or nonwords. In the perceptual task, participants were asked to press a button any time they saw a coloured letter within a string. In the semantic task, participants had to press a button any time they read a word referring to an animal (no letter strings contained a coloured letter). All stimuli were presented for 1.5 s in the centre of the screen. The tasks were presented in different functional runs that were counterbalanced between participants.

For each task, the stimuli included 80 words, of which 40 were high frequency words and 40 were low frequency words, and 80 nonwords. Thus, two sets of words and corresponding nonwords were created and their use in the perceptual task (i.e., low reading demand) and the semantic task (i.e., high reading demand) was counterbalanced between subjects. In both sets, low-frequency words were nouns with a Zipf measure^5^ lower than 4, and high-frequency words were nouns above this cutoff. All word measures were obtained from *EsPal*^33^, and the two sets of words were matched on frequency, length (i.e., 5–8 characters) and number of orthographic neighbours. Nonword strings were included as stimuli in the experimental design to address other research questions not relevant for the present work. To reduce the potential reading demands imposed by nonwords as much as possible, they were designed so that they were legal, legible strings. As Spanish is an orthographically transparent language, this design meant that all nonwords inherited this feature. Furthermore, the two sets of nonwords were also matched in character length. Additionally, we included 13% of Go trials (i.e., either words with a colored letter or animal words) as catch trials for each of the two reading tasks. Nonwords and Go trials were modelled as regressors of interest but not analysed. The stimuli used for the perceptual and semantic reading tasks were also counterbalanced between subjects.

### fMRI data acquisition

Whole-brain fMRI data were obtained on a 3-T Siemens TRIO whole-body MRI scanner (Siemens Medical Solutions) at the Basque Center on Cognition, Brain and Language (BCBL), using a 32-channel whole-head coil. The area between the participants’ heads and the coil was padded with foam in order to reduce head movement, and the participants were asked to stay as still as possible. Snuggly fitting headphones (MR Confon) were used to dampen background scanner noise and to allow communication between participants and experimenters.

The functional images were acquired using a gradient-echo echo-planar pulse sequence with the following parameters: time repetition (TR) = 2000 ms, time echo (TE) = 25 ms, 35 contiguous 3-mm axial slices, 0-mm inter slice gap, flip angle = 90°, field of view = 218 mm, 64 × 64 matrix. The first four volumes of each scan were discarded to allow T1-equilibration effects. The order of the conditions of the study within each run, as well as the inter-trial intervals of variable duration, were determined with an algorithm designed to maximise the efficiency of the recovery of the blood oxygen level-dependent response: Optseq II^34^. High-resolution T1-weighted anatomical images were also acquired with the following acquisition parameters: TR = 2300 ms, TE = 2.97 ms, flip angle = 9°, Field of view = 256 mm, 176 volumes per run, voxel size = 1 cubic mm.

### fMRI data analysis

Standard SPM12 (Wellcome Department of Cognitive Neurology, London, UK) preprocessing routines and analysis methods were employed. Images were corrected for differences in timing of slice acquisition and realigned to the first volume by means of rigid-body motion transformation. Motion parameters were extracted from this process and were used, after a partial smoothing of 4-mm full width at half-maximum (FWHM) isotropic Gaussian kernel, to inform additional motion correction algorithms implemented by the Artifact Repair toolbox (ArtRepair; Stanford Psychiatric Neuroimaging Laboratory), intended to repair outlier volumes with sudden scan-to-scan motion exceeding 0.5 mm and volumes whose signal fluctuations in global intensity was > 1.3% SD away from the mean. The correction of these outlier volumes was performed via linear interpolation between the nearest non-outlier time points^35^. Data from 1 subject requiring more than 15% of their volumes to be repaired was discarded. For the final sample of participants, the average percentage of repaired volumes was 1.8% (SD = 2.8%). After volume repair, functional volumes were co-registered to the T1 images using 12-parameter affine transformation and spatially normalised to the Montreal Neurological Institute (MNI) space by applying nonlinear transforms estimated by deforming the MNI template to each individual’s structural volume. During normalisation, the volumes were sampled to 3-mm cubic voxels. Functional volumes were then smoothed with a 7-mm FWHM isotropic Gaussian kernel. Due to the quadratic relation between separate smoothing operations, the total smoothing applied to the functional data was approximately equivalent to smoothing with an 8-mm FWHM Gaussian kernel. Finally, time series were temporally filtered to eliminate contamination from slow frequency drift (high-pass filter with a cutoff period of 128 s).

Statistical analyses were performed on individual participant data using the general linear model (GLM). fMRI time series data were modelled by a series of impulses convolved with a canonical hemodynamic response function (HRF). The motion parameters for translation (i.e., x, y, and z) and rotation (i.e., yaw, pitch, and roll) were included as covariates of non-interest in the GLM. Each trial was modelled as an event, time-locked to the onset of the presentation of each character string. The resulting functions were used as covariates in a GLM, along with a basic set of cosine functions that high-pass filtered the data. SPM12 FAST was used for temporal autocorrelation modeling in this GLM due to its optimal performance in terms of removing residual autocorrelated noise in first-level analyses (Olszowy et al., 2019)^36^. The least-squares parameter estimates of the height of the best-fitting canonical HRF for each study condition were used in pairwise contrasts. Regarding such analyses, whole-brain contrasts were computed by performing one-sample t-tests on the contrast images.

Region-of-interest (ROI) analyses were carried out by using the MARSBAR toolbox for SPM12^37^. Six left-lateralised regions along the reading network were functionally identified using two different procedures: (I) group level and (II) individual-subject level. The group ROI identification procedure identified active voxels obtained from the whole brain contrast Words > Null across all participants, cluster Family-wise error (FWE) corrected, *p* < 0.001 voxel extent. The regions identified included *pars orbitalis* (centre of mass: − 37, 27, − 8; mm^3^ = 1656), *pars triangularis* (centre of mass:  − 46, 27, 14; mm^3^ = 12,704), *pars opercularis* (centre of mass: − 48, 11, 20; mm^3^ = 5856), MTG (centre of mass: − 46, − 59, − 2; mm^3^ = 1008), IPC (centre of mass: − 30, − 53, 46; mm^3^ = 2200) and vOTC (centre of mass: − 43, − 59, − 17; mm^3^ = 6296). As for the individual ROIs procedure, the same six regions were localised at the individual-subject level. To this end, 5 mm radius spheres were created by selecting the local maxima in each individual subject’s Words > Null contrast (cluster FWE corrected, *p* < 0.001 voxel extent) that fall within the anatomical mask of the six above-mentioned ROIs. For those subjects that had no voxels over the threshold that fell within the anatomical mask, the closest local maxima that allowed a sphere to be built falling within the mask was selected. The selection of the local maxima for individual ROIs in all participants were systematically checked by authors A.S. and *P*.M.*P*-A. The coordinates of each individual ROI per subject can be found in Supplementary Data. For a general overview of the distribution of these coordinates, see also Supplementary Fig. S1.

For functional connectivity analyses, we used the beta-series correlation method^38^ by using custom Matlab scripts for SPM12. Similarly to ROI analyses, functional connectivity analyses were performed on both group and individual ROIs. The occurrence of each event was modelled with the canonical HRF, which allowed for the extraction of the parameter estimates (i.e., beta correlations) associated with each condition in every voxel. Following this, pairwise connectivity between the 6 left-lateralised ROIs described above was conducted. After Bonferroni’s correction, a value of r > 0.355 was considered to show a significant functional connectivity between nodes. Further contrasts (i.e., t-tests) on the beta correlations associated with low versus high Frequency, and perceptual versus semantic Task, were carried out after Fisher’s Z transforms^39^ of the beta Pearson’s r correlations values to make the null hypothesis sampling distribution approach that of the normal distribution.

## Results

### Behavioural performance

Overall, participants showed a high response rate to Go trials, with an average accuracy of 99.4% (SD = 0.02%) for the perceptual task, and 95.4% (SD = 0.05%) for the semantic task. This indicates that participants were focused on the instructions given by the experimenter and performing the task. As expected, accuracy was slightly, but significantly higher for the perceptual task (t = 4.880; *p* < 0.001). Likewise, on average, response times were significantly faster for the perceptual than for the semantic task (t = − 8.697; *p* < 0.001; perceptual M = 560 ms, SD = 90 ms; semantic M = 760 ms, SD = 140 ms).

### Whole-brain contrasts

**Figure 1:**
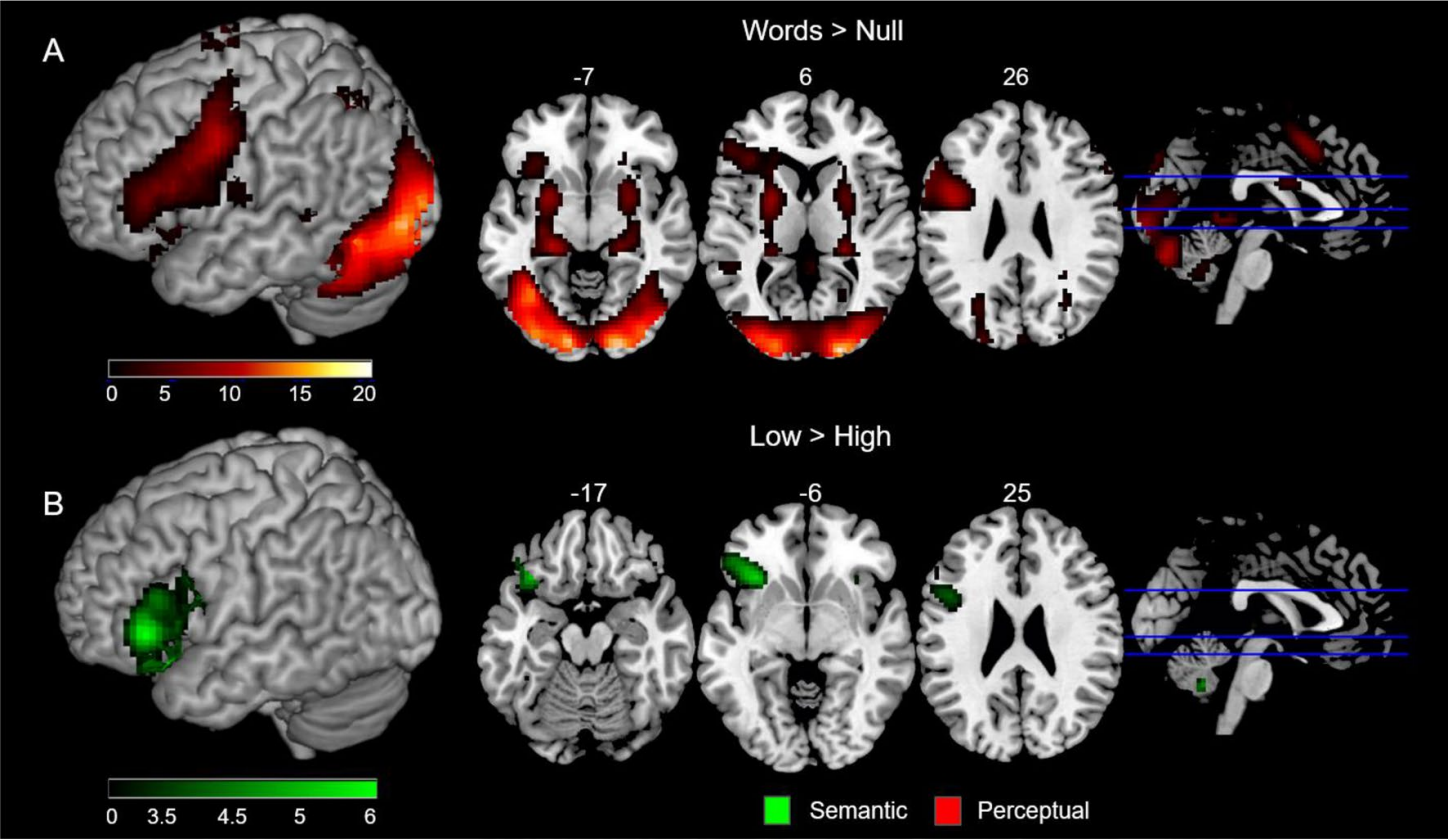
Whole-brain contrasts and low–high frequency simple contrasts. (**A**) Results of the Words *versus* Null contrast across all subjects; (**B**) Low > High frequency contrast in the semantic (high reading demand) task (in green) and in the perceptual (low reading demand) task (in red), *p* < 0.05 FWE corrected clusterwise (*p* < 0.001 uncorrected voxel-extent threshold). No clusters survived this threshold in the perceptual task.

When contrasting all trials including words against baseline, the averaged activation map for words across all subjects (see Fig. 1A) included regions in the occipital cortex, such as the lingual gyrus and the cuneus, the vOTC, IPC, MTG, the middle and superior frontal gyrus, the precentral and postcentral gyri, and the different subregions within the IFG (*pars orbitalis, pars triangularis and pars opercularis*).

In addition, we computed the whole-brain Low > High frequency contrasts in both the perceptual and the semantic task separately, which reflects the brain distribution of the WFE in either task (Fig. 1B). In the perceptual task, no voxels survived the threshold of *p* < 0.05 (FWE corrected clusterwise, with a *p* < 0.001 uncorrected voxel-extent threshold). In contrast, the semantic task showed a WFE exceeding the established threshold in the whole left IFG, with global maxima located in the *pars triangularis* and *pars orbitalis*.

### ROI analyses

**Figure 2:**
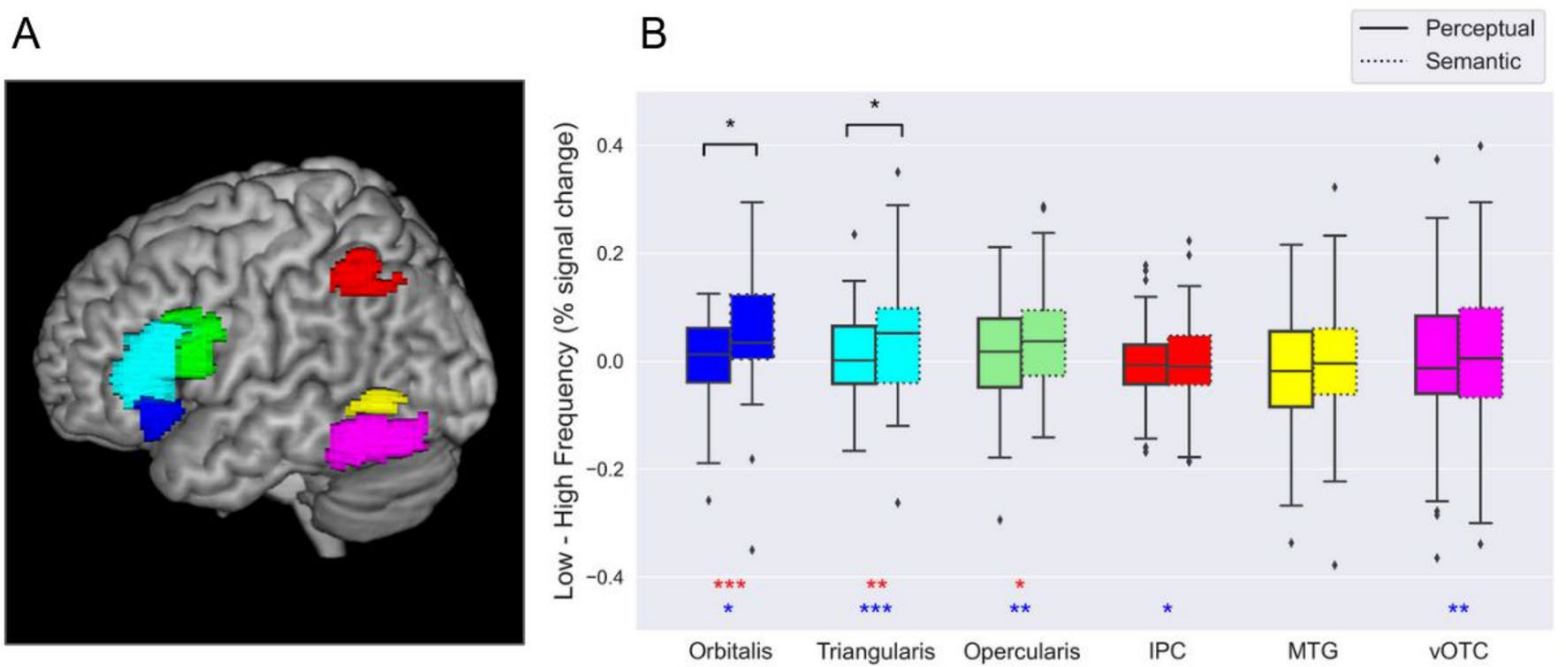
ROI analyses. (**A**) Group ROIs employed, obtained from the Words-Null contrast. (**B**) Results from the group ROI analyses. ROIs are represented in the X axis, whereas the difference of the parameter estimates between regional activation of low *versus* high frequency words is depicted in the Y axis. Boxes with straight lines represent the perceptual task, whereas boxes with dotted lines depict the semantic task. Red asterisks at the bottom part indicate that the region showed a significant main effect of Frequency (*p < .05, BF > 1; **p < .01, BF > 5; ***p < .001, BF > 10). Blue asterisks at the bottom part indicate that the region showed a significant main effect of Task (*p < .05, BF > 1; **p < .01, BF > 5; ***p < .001, BF > 10). Black asterisks over the boxes indicate that the region showed a significant Frequency x Task interaction, due to a significant difference in the WFE between the perceptual and the semantic tasks (*p < .05). Supplementary Fig. S2 shows the same group ROI results with separated boxes for low and high frequency % signal change.

For each of the selected ROIs, a 2 × 2 ANOVA with Frequency and Task as factors, and percent signal change (PSC) as the dependent measure, was carried out. Results from group ROIs are reported first, followed by individual ROIs results These results from the group and individual ROI ANOVAs are summarized in Table 2, which shows main effects of Frequency and Task and the Frequency x Task interaction. Below, we highlight the results from the simple post-hoc pairwise t-tests for planned comparisons, for which Bayes factors (BF) are also reported. Figure 2 shows an overview of the results regarding the WFE by Task, across all group ROIs. An additional 2 × 2x2 ANOVA, with ROI type (group *versus* individual ROI) x Frequency x Task was carried out to determine any possible effects arising from the type of ROIs used. Aside from a significant ROI type x Task interaction in IFG *pars triangularis* (F = 8.078, *p* = 0.004)*,* we found no significant main or interactive effects of the factor ROI type and, therefore, only results from group ROIs are shown in Fig. 2.

**Table 2:** Statistical results from the separate group and individual ROI ANOVAs. Main effects of Frequency and Task, as well as their interaction are reported. Asterisks denote a statistically significant effect.

ROI names	Group ROI			Individual ROIs		
	Frequency	Task	Interaction	Frequency	Task	Interaction
Orbitalis	F = 10.347	F = 7.222	F = 5.595	F = 7.058	F = 5.870	F = 3.211
	*p* = 0.002*	*p* = 0.009*	*p* = 0.021*	*p* = 0.010*	*p* = 0.018*	*p* = 0.079
	${\eta }^{2}$= 0.163	${\eta }^{2}$= 0.119	${\eta }^{2}$= 0.095	${\eta }^{2}$= 0.121	${\eta }^{2}$= 0.103	${\eta }^{2}$= 0.059
Triangularis	F = 6.043	F = 11.946	F = 4.812	F = 4.710	F = 43.873	F = 2.785
	*p* = 0.017*	*p* = 0.001*	*p* = 0.017*	*p* = 0.034*	*p* < 0.001*	*p* = 0.101
	${\eta }^{2}$= 0.102	${\eta }^{2}$= 0.183	${\eta }^{2}$ = 0.083	${\eta }^{2}$= 0.083	${\eta }^{2}$= 0.457	${\eta }^{2}$= 0.050
Opercularis	F = 5.384	F = 9.345	F = 1.957	F = 8.035	F = 20.109	F = 0.835
	*p* = 0.024*	*p* = 0.003*	*p* = 0.16	*p* = 0.006*	*p* < 0.001*	*p* = 0.364
	${\eta }^{2}$= 0.092	${\eta }^{2}$= 0.149	${\eta }^{2}$ = 0.035	${\eta }^{2}$= 0.133	${\eta }^{2}$= 0.278	${\eta }^{2}$= 0.015
IPC	F = 4.425	F = 0.309	F = 0.012	F = 4.022	F = 0.134	F = 0.596
	*p* = 0.443	*p* = 0.040*	*p* = 0.580	*p* = 0.715	*p* = 0.050*	*p* = 0.715
	${\eta }^{2}$= 0.011	${\eta }^{2}$= 0.077	${\eta }^{2}$ = 0.005	${\eta }^{2}$< 0.001	${\eta }^{2}$= 0.071	${\eta }^{2}$= 0.002
MTG/STG	F = 0.367	F = 2.169	F = 0.008	F = 0.077	F = 0.972	F = 0.023
	*p* = 0.547	*p* = 0.146	*p* = 0.928	*p* = 0.781	*p* = 0.328	*p* = 0.879
	${\eta }^{2}$= 0.006	${\eta }^{2}$= 0.039	${\eta }^{2}$< 0.001	${\eta }^{2}$ = 0.001	${\eta }^{2}$= 0.018	${\eta }^{2}$< 0.001
vOTC	F = 0.013	F = 7.331	F = 0.725	F = 0.060	F = 9.841	F = 1.704
	*p* = 0.909	*p* = 0.009*	*p* = 0.398	*p* = 0.807	*p* = 0.002*	*p* = 0.197
	${\eta }^{2}$< 0.001	${\eta }^{2}$= 0.121	${\eta }^{2}$ = 0.013	${\eta }^{2}$= 0.001	${\eta }^{2}$ = 0.159	${\eta }^{2}$ = 0.031

*Pars orbitalis.* Following the group ROI approach, a main effect of Frequency was found for this region. We also found a significant main effect of Task. These main effects were qualified by a significant Frequency x Task interaction. Simple-effect post-hoc analyses revealed that this interaction was due to low-frequency words showing stronger regional activation than high-frequency words in the semantic (t = − 3.907, *p* < 0.001, BF = 91.709), but not in the perceptual task (t = − 0.687, *p* = 0.495, BF = 0.186).

Results from the group ROI of the *pars orbitalis* were replicated with individual ROIs, although the Frequency x Task interaction resulted marginally significant, possibly due to differences in signal intensities derived from both approaches.

*Pars triangularis.* Following group ROI analysis, a main effect of Frequency was found in this region. We also found a statistically significant main effect of Task. Moreover, these main effects were qualified by a statistically significant Frequency x Task interaction, which was again due to low-frequency words showing stronger regional activation than high-frequency words in the semantic (t = − 2.860, *p* = 0.006, BF = 5.656), but not in the perceptual task (t = − 0.486, *p* = 0.627, BF = 0.228).

The individual ROI approach replicated the results from the group ROI approach, but the Frequency x Task interaction was just statistically marginal in the individual *pars triangularis* ROI analysis. As mentioned above, when including the ROI type as a factor in the ANOVA, we found a significant ROI type x Task interaction in this region (F = 8.078, *p* = 0.004).

*Pars opercularis*. The ANOVA for this group ROI revealed a statistically significant main effect of Frequency, driven by low-frequency words showing higher regional activation than high-frequency words (t = − 2.320, *p* = 0.024, BF = 1.722). Moreover, there was a statistically significant main effect of Task, due to regional activation being overall stronger in the semantic than in the perceptual task (t = − 3.057, *p* = 0.003, BF = 9.117). No statistically significant Frequency x Task interaction was found.

The individual ROIs approach replicated these results.

*IPC*. Following the group ROI approach, no main effect of Frequency was found in this region. The main effect of Task was statistically significant and it was due to regional activation being stronger in the semantic than in the perceptual reading task (t = − 2.103, *p* = 0.040, BF = 1.131). No statistically significant Frequency x Task interaction was found.

The individual ROIs analysis replicated this main effect.

*MTG*. With the group ROI, we found no statistically significant main effects of Frequency or Task in this region. No statistically significant Frequency x Task interaction was found either.

The individual ROIs analysis replicated these results.

*vOTC*. vOTC group ROI revealed no statistically significant main effect of Frequency, but there was a statistically significant main effect of Task. As in other regional analyses, this effect was due to activation being stronger in the semantic than in the perceptual reading task (t = − 2.793, *p* = 0.007, BF = 4.828). The Frequency x Task interaction was not statistically significant.

The same pattern of results was obtained with individual vOTC ROIs.

### Functional connectivity analyses

**Figure 3:**
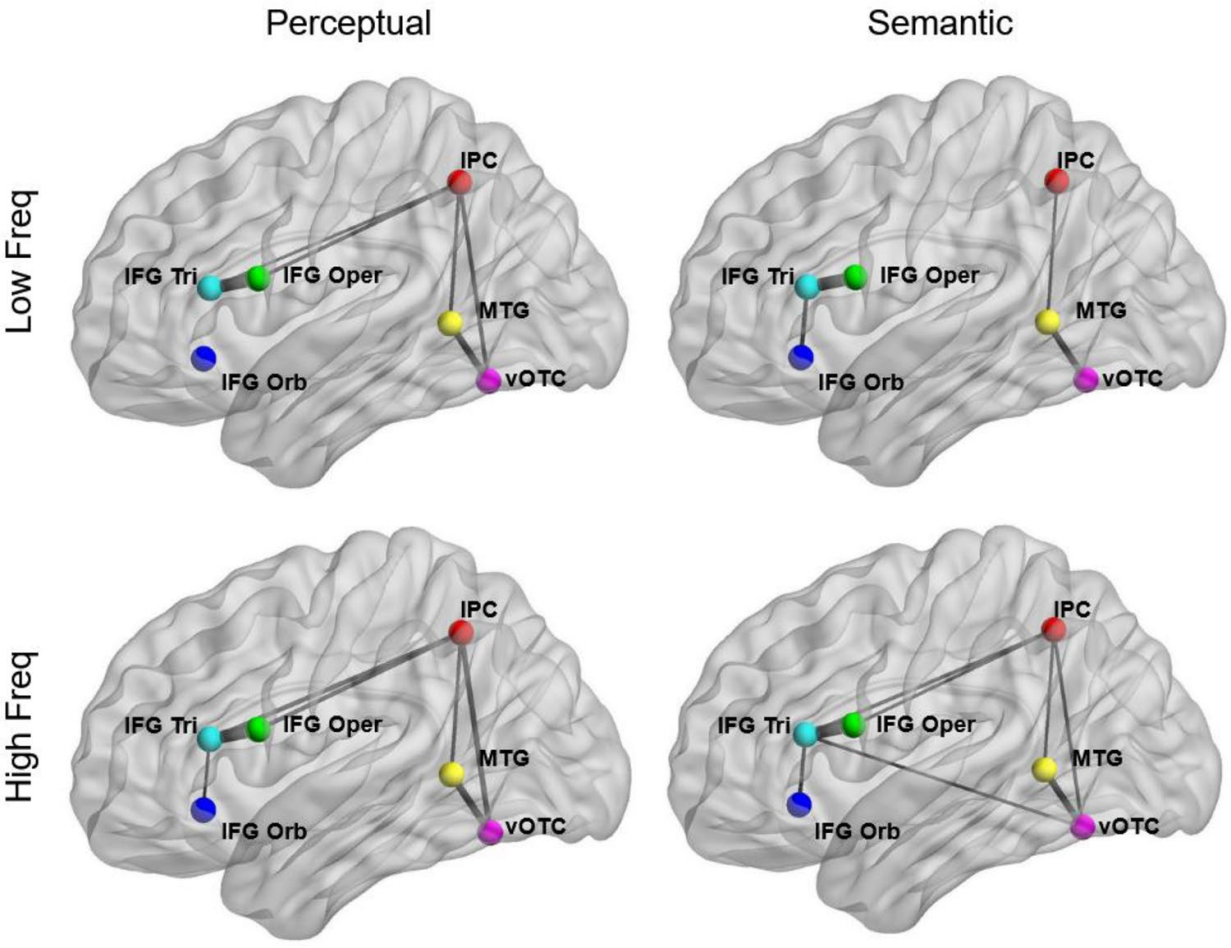
Pairwise connectivity between all the 6 group ROIs. Edges were drawn if they exceeded the threshold of r = 0.355, which was the estimated threshold for a pair of nodes to be significantly co-activated, after Bonferroni correction. Thicker edges represent stronger beta-correlation values.

Functional connectivity analyses reproduced previous evidence demonstrating the relevance of the dorsal and ventral networks in reading^18,19^. Following the group ROIs analytical approach, overall across conditions we found tight coupling between IFG regions: *pars triangularis*—*pars orbitalis* (r = 0.39, *p* < 0.01) and *pars opercularis*—*pars triangularis* (r = 0.76, *p* < 0.001). vOTC showed significant connectivity with MTG (r = 0.53, *p* < 0.001) and IPC (r = 0.40, *p* < 0.01), which in turn showed stronger coupling with MTG (r = 0.40, *p* < 0.01), IFG *pars opercularis* (r = 0.38, *p* < 0.01) and *pars triangularis* (r = 0.39, *p* < 0.01). However, we found no statistically significant effects of word frequency or reading demands on functional connectivity, when following both group and individual ROIs approaches. Figure 3 illustrates the pairwise functional connectivity among all group ROIs as a function of Frequency and Task.

## Discussion

We investigated the effects of word frequency on the activation and coactivation of language networks, by using complementary fMRI analytical approaches. Additionally, we assessed the interaction between frequency effects and reading demands. We found that word frequency alone influenced the regional engagement of the left IFG *pars opercularis*, whereas reading demands separately modulated the regional engagement of the IFG *pars opercularis,* IPC and vOTC. Most importantly, we found an interaction effect in anterior IFG regions (i.e., *pars orbitalis* and *pars triangularis*) with word frequency being modulated by reading demands only in the semantic task. Although significant functional coupling among nodes of the reading networks was observed, there were no differences in functional connectivity as a function of word frequency or reading demands. These main results are discussed below.

### Word frequency

The IFG in general has been repeatedly found to respond to word frequency^7,9,10,12,13,15,16,40^. In terms of regional activation, the left IFG *pars opercularis* was the only region that showed a WFE per se, not influenced by reading demands, consistent across group and individual ROIs analytical approaches. Some previous interpretations attributed this effect in the *pars opercularis* to phonological processing during reading tasks^7,15^. Under this view, the rapid availability of high-frequency words makes them require less phonological mediation than low-frequency words, which explains the higher engagement in this region for low versus high frequency words^15^. Our results are in line with the view that engagement of the *pars opercularis* underlies WFE effects that arise due to differences in phonological processing costs.

### Reading demands

Several regions in our study showed differential patterns of regional activation as a function of reading demands. The left *pars opercularis* consistently showed higher regional activation in the semantic as compared to the perceptual reading task. This effect could possibly be due to stronger processing demands in this anterior IFG region during semantic reading and, more specifically, to the fact that the high reading demand task also involves phonological processing, consistent with a wealth of evidence highlighting the role of this region in processing phonological aspects. A similar reading demand effect was observed in the IPC. In fact, both the *pars opercularis* and IPC are considered to be part of the phonological dorsal reading network and their activation profile in this study could also reflect phonological processing during lexical search (e.g.,^18,41^).

In the present study, the vOTC activation pattern was related to task demand but not word frequency. Although some previous studies have reported an effect of word frequency over regional activation in the vOTC^9,10,12–14,16,17^, other studies also failed to find such an effect^7,11^. One possible factor contributing to mixed results in the vOTC in this regard is the variety of reading tasks used in these studies and the consequent differences in task demands. Among the studies finding a WFE in vOTC, the tasks used range from passive reading with no specific instructions^9,10,13^, to overt naming^12^, or semantic tasks based on the visual/conceptual features of the object represented by the stimulus words^17^. On the other hand, studies using lexical decision tasks failed to find this effect in the vOTC^7,11^. To our knowledge, only one study analysing the neural correlates of word frequency actively manipulated reading demands^6^. In this study, the authors included a similar manipulation, finding a WFE in vOTC only in a task involving semantic judgements relative to a passive reading task.

Some authors have argued that a part of the vOTC, also named as visual word form area (VWFA), is dedicated to pre-lexical processing of visual word forms^23^. According to this view, the VWFA activation reflects prelexical processing of visual features of words, and the heightened activation patterns are due to long exposure times or the influence of top-down processes. These authors have typically used low-demand reading tasks that do not require any sort of semantic processing (e.g. indicating whether a stimulus was presented twice or not)^42^. The finding that the vOTC also responds to non-word visual stimuli has led to the idea that the vOTC is not exclusively dedicated to the pre-lexical processing of visual word forms^24^. This view states that the vOTC integrates bottom-up visual information and top-down expectations in order to process visual features^20^, in line with studies showing that different regions within the vOTC are involved in these processes^29^. Authors favouring this view have typically used higher-demand tasks that require some level of semantic processing (e.g., picture naming)^43^. In the present study, we actively manipulated reading demands, and we found that the vOTC is differentially involved in word reading as a function of the demands imposed by the task, which is consistent with the view that the vOTC integrates both bottom-up and top-down processes.

### The WFE is modulated by reading demands in anterior IFG

In the present study, we found an effect of word frequency that was modulated by reading demand in *pars orbitalis* and *pars triangularis*. In both of these regions, the WFE was only present in the semantic (high reading demand), but not in the perceptual (low reading demand) task. Thus, word frequency influenced regional activation only when participants had to actively retrieve word meaning to perform the task. This may reflect the role of anterior IFG in controlling access to previously stored semantic representations^44^, and/or in the unification and manipulation of semantic information^1,45^. These findings strengthen the view that specifically the anterior IFG plays an active role in the controlled access to semantic information^6,44,46^, while the posterior IFG is engaged in phonological processing during lexical search^7,15^. Moreover, this finding supports the idea that the anterior part of the IFG is involved in deliberate access to semantics, rather than retrieval effort alone^6^. In line with our prediction, these results underline the involvement of the lexico-semantic ventral reading network in the WFE.

### Task-related functional connectivity

A different pattern of results was found regarding functional coactivation between nodes in the language networks examined here as a function of our experimental design. To our knowledge, this is the first study examining the functional connectivity patterns associated with the WFE during reading tasks. In line with previous findings from regional activation analyses, we hypothesised that low-frequency words, especially in the semantic task, would show a pattern of stronger functional connectivity between nodes within the ventral reading network. Nevertheless, our results do not indicate any effects of frequency on functional connectivity. We just found that both networks were active during reading, but we observed no differences as a function of word frequency or task demands. A possible explanation for the absence of frequency or task effects at the level of functional connectivity might be that these effects are resolved at the regional level, and thus no differential functional connectivity patterns emerged from such effects. This interpretation is in line with previous evidence in the field of reading deficits, where specific effects arise at the level of regional activation, but such pattern of results is not observed in functional connectivity^41^. These studies illustrate that different mechanisms of brain reorganisation may rely either on regional effects or functional connectivity patterns^47^. However, as this is the first study analysing possible functional connectivity patterns underlying the WFE, this result should be interpreted cautiously, and future studies are required to replicate these results.

### Limitations

Finally, two limitations of the present study should be mentioned. Firstly, we performed only mass univariate analyses. These can reveal if any region is (putatively) taking part in any effect, such as the IFG in the WFE. Multivariate designs are better suited for exploring how a brain region represents information. For this reason, future studies examining the effect of psycholinguistic variables such as word frequency should include multivariate designs. Secondly, we used a categorical definition of word frequency, which does not fully represent the continuum of word frequency. Furthermore, other psycholinguistic variables, such as word familiarity or word concreteness, are known to interact with word frequency. Future investigations should be based on designs that include a wider variety of psycholinguistic variables known to play a relevant role in lexical representations, as well as a finer definition (e.g., continuous definitions) of such variables.

## Conclusions

By applying different analytical approaches to a large dataset of 54 individuals, here we offer robust evidence that the activity in the left *pars opercularis* is modulated by word frequency, possibly reflecting phonological processing during lexical search. The activation of the *pars opercularis* and IPC are also modulated by reading demands, possibly reflecting stronger phonological processing during semantic word reading. The same reading demand effect was observed in the vOTC, but in the absence of any effect of word frequency, which seems to support the notion that this area is influenced by top-down processes. This has potential implications for theories about the role of the vOTC in pre-lexical and lexical processes. Finally, the WFE is modulated by reading demands in *pars orbitalis and pars triangularis*, since the WFE was only present under semantic reading demands. This is interpreted as an indicator of the role of the anterior IFG in controlled access to semantics. These effects, occurring at the regional activation level, seem to underline the role of the ventral reading network in the access to lexico-semantic information.

### Supplementary Information

Supplementary Information 1.

Supplementary Information 2.

## Data Availability

The fMRI data that support the findings of this article are available to researchers via the following data access procedure: https://www.bcbl.eu/Datasharing/ScientificReports2023Sanchez/.
