# Supplementary material for: Word frequency and reading demands modulate brain activation in the inferior frontal gyrus
Source: Sci Rep. 2023 Oct 11;13:17217. doi: 10.1038/s41598-023-44420-z (PMC10567770; doi:10.1038/s41598-023-44420-z)
Supplement: Supplementary file 2 — Supplementary Information 2. [file 41598_2023_44420_MOESM2_ESM.docx]

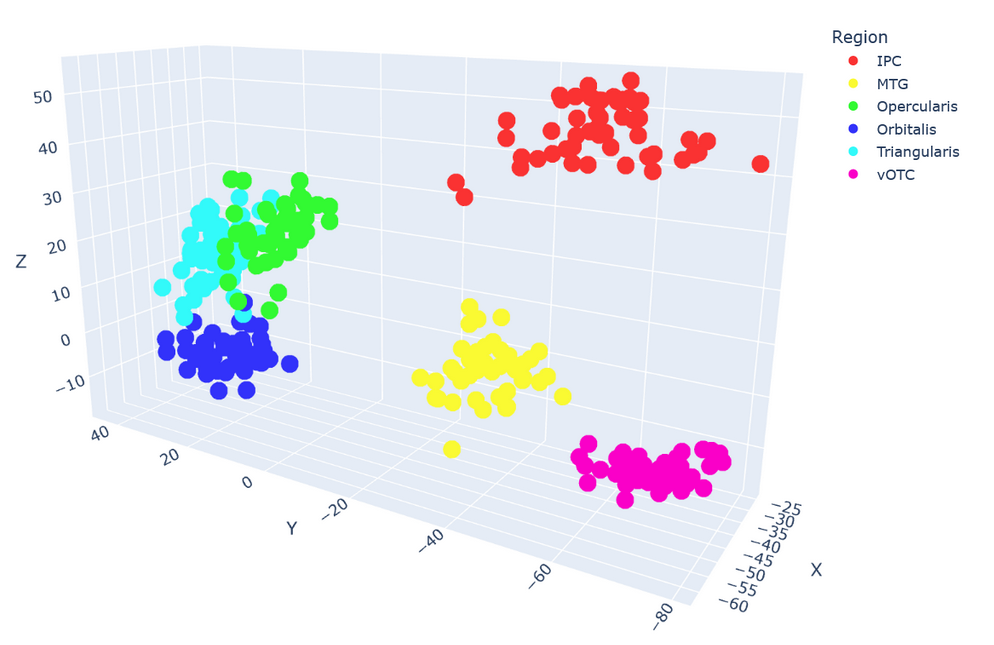


Figure S1. Distribution of the individual ROIs in the X Y Z MNI space. Each dot represents a participant.


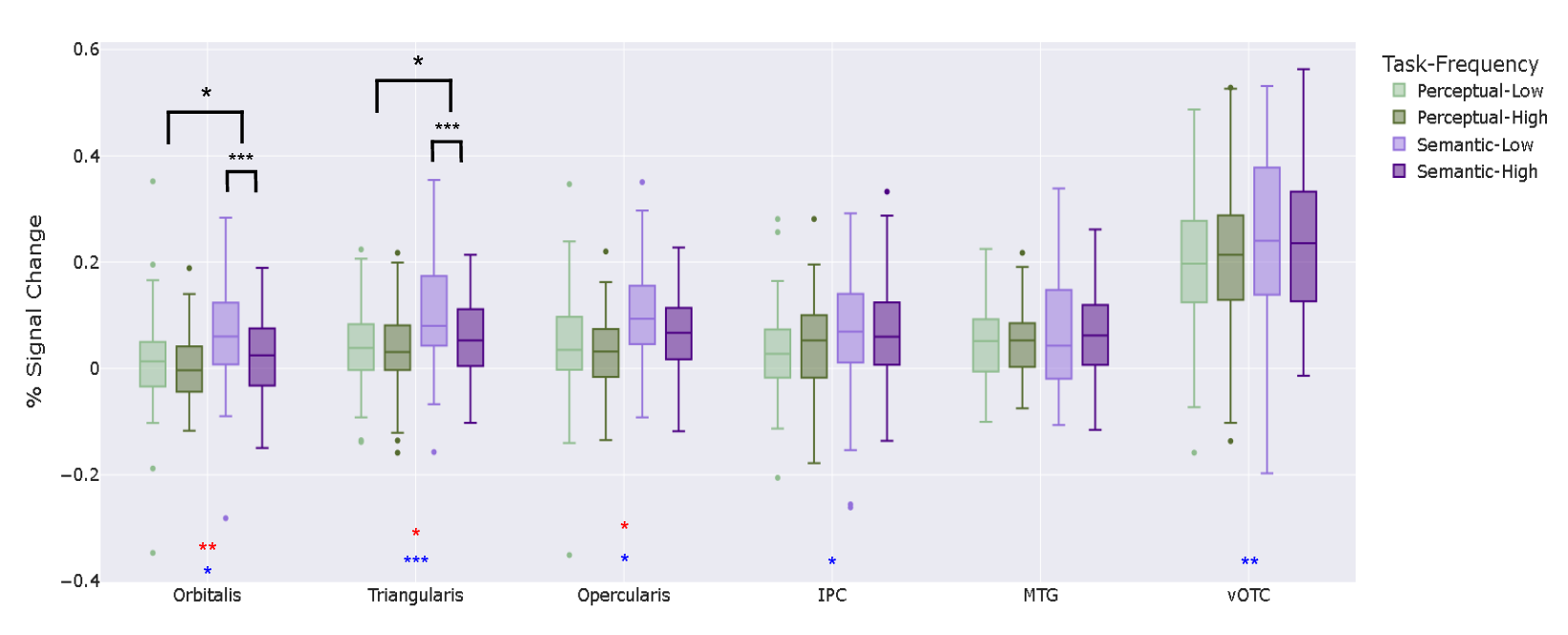


Figure S2. Percent signal change in each of the two tasks, for Low vs. High Frequency words. As in Figure 2*B*, red asterisks denote a main effect of Frequency (*p < .05, BF > 1; **p < .01, BF > 5; ***p < .001, BF > 10). Blue asterisks indicate a main effect of Task (*p < .05, BF > 1; **p < .01, BF > 5; ***p < .001, BF > 10). Black asterisks indicate a significant Task x Frequency interaction (*p < .05, BF > 1; **p < .01, BF > 5; ***p < .001, BF > 10).
